# Validation of a high‐fidelity simulator for diagnostic knee arthroscopy training

**DOI:** 10.1002/jeo2.70684

**Published:** 2026-03-18

**Authors:** Oliver Tze Rong Lim, Jeremy Tze En Lim, Jingyan Fang, Dileepa Banagala, Samundeeswari Saseendar, Salim A. L. Habsi, James Hui, Yee Han Dave Lee, Ying Ren Mok

**Affiliations:** ^1^ Yong Loo Lin School of Medicine National University of Singapore Singapore; ^2^ Department of Orthopaedic Surgery Singapore General Hospital Singapore; ^3^ Department of Orthopaedic Surgery National University Hospital Singapore

**Keywords:** construct, content, face, simulator, validity

## Abstract

**Purpose:**

To validate a high‐fidelity arthroscopic simulator by assessing its construct, face and content validity for diagnostic knee arthroscopy training.

**Methods:**

Eighty‐one participants (novices = 41, intermediates = 20 and experts = 20) performed a knee arthroscopy navigation task requiring identification of nine intra‐articular landmarks, repeated for up to five trials with a 10‐min limit per trial. Task‐completion times and attrition were recorded. Performance was analysed using Kruskal–Wallis and Mann–Whitney tests with effect sizes. Post‐task questionnaires evaluated face validity, content validity, educational value and user‐friendliness.

**Results:**

Performance times differed significantly between experience groups (*p* < 0.001–0.043). Experts completed Trial 1 in roughly one third the time required by novices (90 s vs. 292 s; *r* = 0.78). All groups improved with repetition, with steep early gains in novices and the most consistent improvement among intermediates. Between‐group differences narrowed in later trials, although interpretation is limited by attrition and survivorship bias, with only six novices completing Trial 5. Face validity ratings were high (mean > 7/10), and content validity was strongly endorsed (92%–97% supporting curricular integration). Educational value was rated positively (92.3–97.4%), whereas user‐friendliness received lower agreement (35.9%).

**Conclusions:**

This high‐fidelity simulator demonstrated strong construct, face and content validity, reliably discriminating between experience levels and supporting early‐phase skills acquisition. Strong realism and educational value ratings support its potential role in structured arthroscopic training, although usability findings highlight opportunities for ergonomic and instructional refinement. Future research should assess transfer validity and operative performance impact.

**Level of Evidence:**

Level III, prospective observational validation study.

**Abbreviations**
ACL: anterior cruciate ligament
IQR: interquartile range
IRB: institutional review board
NRS: numerical rating scale
SD: standard deviation
VR: virtual reality

## INTRODUCTION

Arthroscopic surgery is a core technical competency in orthopaedics, yet early operative exposure is limited within traditional apprenticeship training, where observation seldom provides structured deliberate practice. The procedure requires precise hand–eye coordination, triangulation through constrained joint spaces, and interpretation of indirect visual fields, contributing to a steep learning curve and risk of iatrogenic error. Simulation has therefore become an important adjunct for skill acquisition, enabling repetitive practice of instrument handling and anatomical navigation in a risk‐free environment.

A variety of simulator modalities exist, including virtual‐reality (VR) platforms [8, 11], low‐fidelity bench models [1], hybrid systems blending visual and tactile components [6, 13], and physical simulators emphasising realistic haptics and tissue‐instrument interaction [5, 10]. VR simulators offer automated metrics and procedural variability but often lack robust haptic feedback, whereas physical simulators better replicate resistance, bimanual triangulation, and tactile decision‐making [4, 12]. Systematic reviews and meta‐analyses demonstrate that orthopaedic simulators improve technical performance and support structured competency‐based curricula [2, 7, 14, 15].

The Tactile Orthopaedics Arthroscopy Knee simulator is a physical high‐fidelity platform designed to enhance realism through dynamic joint positioning and haptic resistance from cartilage, meniscus, and ligament constructs. However, no peer‐reviewed validation studies evaluating its educational effectiveness currently exist; published material is limited to manufacturer documentation. A formal assessment of its validity is therefore necessary. Unlike most previously validated platforms, which are predominantly VR or hybrid systems, this simulator is a fully physical model emphasising haptic resistance and dynamic joint manipulation without reliance on virtual environments.

Validity frameworks commonly used in arthroscopic simulation research assess construct validity (ability to discriminate between experience levels), face validity (perceived realism) and content validity (coverage of essential procedural tasks) [3, 9]. Additional considerations include educational value and user‐friendliness [12], which influence practical adoption in residency programs. To our knowledge, this is the first peer‐reviewed validation study evaluating this simulator platform.

Therefore, the aim of this study was to evaluate the educational effectiveness of the tactile knee simulator by assessing its construct, face and content validity, as well as its perceived educational value and usability for orthopaedic training. Diagnostic navigation was selected as the validation task because it represents the foundational arthroscopic competency upon which all therapeutic procedures depend, particularly during early skill acquisition. We hypothesised that the simulator would discriminate between novice, intermediate and expert users, demonstrate high perceived realism, and adequately represent the essential components of diagnostic knee arthroscopy.

## METHODS

### Study design

This prospective validation study was conducted at a single tertiary institution. Eighty‐one participants were recruited and stratified by prior arthroscopy experience: novices (*n* = 41; 0 cases), intermediates (*n* = 20; 1–59 cases) and experts (*n* = 20; ≥60 cases). Group thresholds were based on previous arthroscopy validation literature, in which ≥60 completed procedures are commonly used to define expert‐level proficiency. All participants provided written informed consent and completed a demographic questionnaire including age, sex, handedness, professional level and prior simulation exposure. Ethics approval was obtained from the Institutional Review Board (approval no. 2024‐3242). Written informed consent was obtained from all participants.

### Simulator task

Participants performed a diagnostic knee navigation task on the tactile knee arthroscopy simulator (Figure 1), locating nine predefined intra‐articular landmarks using standard arthroscopic instrumentation. Up to five repetitions were permitted, with a 10‐minute limit per repetition. The task sequence is summarised in Appendix SA. Task time was defined as the cumulative interval between landmarks. All procedures were video‐recorded and analysed by a single assessor using predefined timing anchors. Blinding was not feasible due to visible technique differences and is acknowledged as a limitation. Diagnostic navigation was selected as it represents a foundational arthroscopic competency underpinning all subsequent therapeutic procedures.

**Figure 1:**
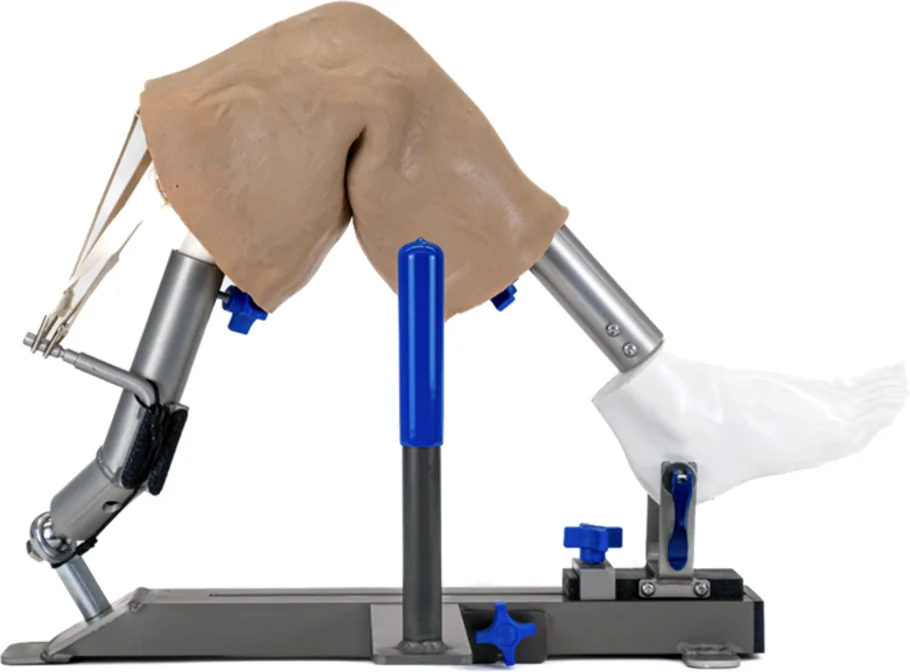
The tactile knee high‐fidelity arthroscopic simulator.

### Pre‐ and post‐study questionnaires

A pre‐study questionnaire recorded demographic information and potential performance‐related variables, including age, gender, handedness, professional role, arthroscopy experience, video‐gaming and sports background, and interest in surgical specialities. After completing the simulator task, participants completed a post‐study questionnaire assessing face validity, content validity, educational value and user‐friendliness using either a 10‐point Likert scale or dichotomous responses. Questionnaire items are presented in Appendix SB. Face and content validity assessments were completed only by intermediate and expert participants (*n* = 40), who had sufficient real‐world exposure to judge realism.

### Statistical analysis

Task‐time data demonstrated right‐skewed distributions with extreme values across groups, justifying the use of non‐parametric statistical tests. Performance differences between experience groups were assessed using Kruskal–Wallis (KW) tests with Mann–Whitney *U* (MWU) pairwise comparisons. Effect sizes were calculated using *η* ^2^ (eta‐squared) for overall group comparisons (KW) and *r* (rank‐biserial correlation) for pairwise contrasts (MWU). Questionnaire responses were summarised descriptively (median, IQR, frequencies). Statistical analysis was performed using R (version 4.2; R Foundation for Statistical Computing). Prior simulator exposure was recorded descriptively but was not included as a covariate due to its low prevalence (7.4%) and limited variability.

Learning curves were analysed descriptively using trial ‐wise performance trends rather than formal curve modelling. Objective motion metrics were unavailable, as the simulator does not incorporate automated instrument‐tracking capabilities. Given the exploratory nature of construct validation, pairwise comparisons were interpreted alongside effect sizes rather than applying formal multiple‐comparison correction. Confidence intervals were not calculated for effect sizes due to the non‐parametric analytic framework and stability of interval estimates.

Analyses were performed using complete‐case data for each trial.

## RESULTS

### Participant demographics

Eighty‐one participants completed the study, comprising 41 novices, 20 intermediates and 20 experts. The mean age was 31.9 years; 65.4% (53/81) were male; and participants included medical students, residents and consultant surgeons. Prior experience with simulation was reported by 7.4% (6/81) of participants. Detailed demographic characteristics are summarised in Table 1.

**Table 1:** Demographic of participants.

	Novices (0 arthroscopies)	Intermediates (1–59 arthroscopies)	Experts (>60 arthroscopies)
Participants, *n*	41	20	20
Male, %	53.7%	75.0%	80.0%
Age, years	27.2	35.0	38.5

### 3.2 Construct validity

Task completion times differed significantly between experience groups across all five trials (*p* < 0.001–0.043; Tables 2 and 3, Figures 2 and 3). In Trial 1, median times were 292 s (novices), 200 s (intermediates), and 90 s (experts), with large effect‐size differences between experts and both other groups (*r* = 0.71–0.78). All groups improved with repetition, with novices showing the steepest time reduction (292 s → 73 s).

**Table 2:** Task time per experience group per repetition (in seconds).

	Novices	Intermediates	Experts
Knee Trial 1	292 (238–390) [41]	200 (158–294) [20]	90 (55–109) [20]
Knee Trial 2	283 (212–355) [37]	127 (111–231) [20]	61 (52–93) [20]
Knee Trial 3	280 (206–311) [31]	114 (82–125) [17]	58 (46–86) [20]
Knee Trial 4	86 (61–113) [14]	87 (74–96) [14]	58 (44–77) [20]
Knee Trial 5	73 (44–140) [6]	78 (75–93) [9]	49 (37–56) [20]

*Note*: Task times are presented as median (interquartile range) [number of participants who completed the trial].

**Table 3:** Statistical comparisons between groups with *p* values and effect sizes (*η* ^2^ for Kruskal–Wallis; *r* for Mann–Whitney *U* tests).

Trial	All three groups	Novice vs. intermediate	Novice vs. expert	Intermediate vs. expert
1	*p* < 0.001, *η* ^2^ = 0.54	*p* = 0.005, *r* = 0.36 (medium)	*p* < 0.001, *r* = 0.78 (large)	*p* < 0.001, *r* = 0.71 (large)
2	*p* < 0.001, *η* ^2^ = 0.63	*p* < 0.001, *r* = 0.57 (medium–large)	*p* < 0.001, *r* = 0.81 (large)	*p* < 0.001, *r* = 0.59 (medium–large)
3	*p* < 0.001, *η* ^2^ = 0.75	*p* < 0.001, *r* = 0.79 (large)	*p* < 0.001, *r* = 0.83 (large)	*p* = 0.003, *r* = 0.49 (medium–large)
4	*p* = 0.043, *η* ^2^ = 0.13	*p* = 0.890, *r* = 0.03 (negligible)	*p* = 0.052, *r* = 0.34 (medium)	*p* = 0.026, *r* = 0.38 (medium)
5	*p* = 0.033, *η* ^2^ = 0.20	*p* = 0.953, *r* = 0.03 (negligible)	*p* = 0.153, *r* = 0.29 (small)	*p* = 0.012, *r* = 0.47 (medium–large)

**Figure 2:**
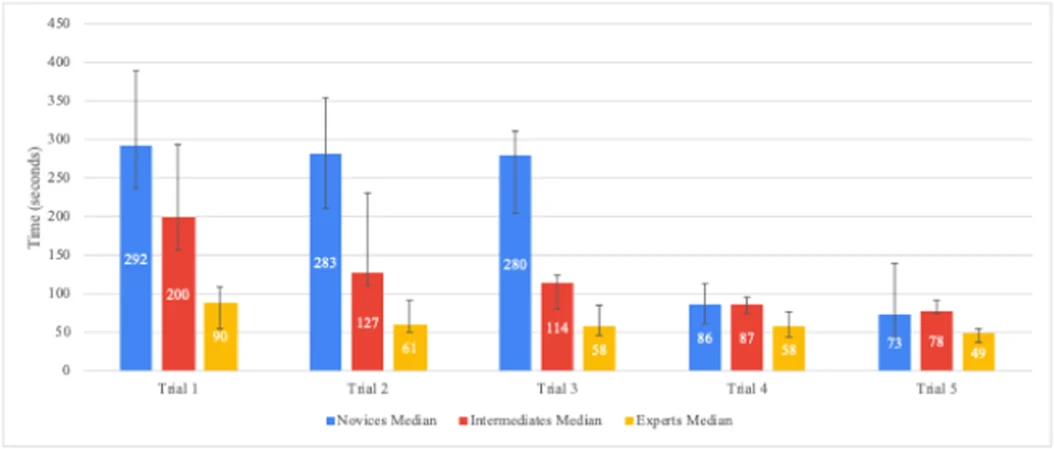
Median task completion times across experience groups.

**Figure 3:**
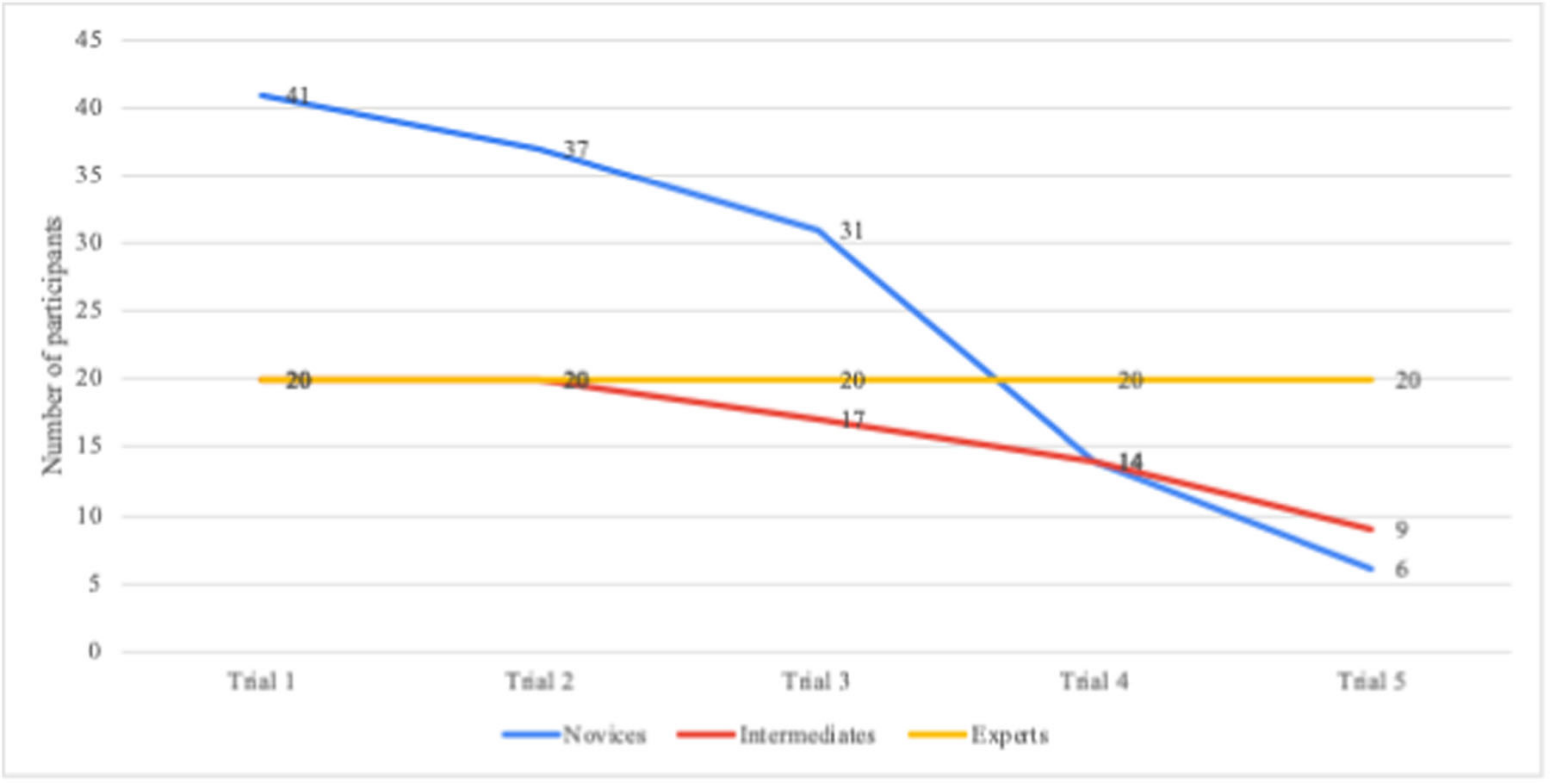
Participant attrition across repeated simulator trials.

Between‐group differences diminished over subsequent trials, with novice–intermediate differences becoming negligible by Trials 4 and 5 (*p* = 0.890–0.953; *r* = 0.03), while expert–intermediate differences remained significant at Trial 5 (*p* = 0.012; *r* = 0.47). However, this convergence should be interpreted cautiously, as only six novices completed Trial 5, reflecting attrition of slower participants rather than true group‐level equivalence. Experts maintained consistently rapid performance (58–49 s) with no attrition, whereas novice and intermediate numbers declined markedly after Trial 3 (Figure 3).

### Face validity

Face‐validity scores were favourable across all domains, with mean visual and anatomical realism scores exceeding 7.0 (Table 4). Highest ratings were observed for joint clarity (9.02) and portal clarity (8.88), while manipulation realism received the lowest score (6.76), suggesting areas for refinement.

**Table 4:** Results of the questionnaire for face validity.

Domain	Mean score (standard deviation)
Outer appearance	8.05 (1.40)
Joint clarity	9.02 (1.06)
Portal clarity	8.88 (1.19)
Intra‐articular realism	7.78 (1.46)
Texture realism	7.32 (1.59)
Colour realism	7.39 (1.74)
Structure size realism	8.00 (1.41)
Joint space size realism	7.59 (1.48)
Image realism	7.83 (1.56)
Manipulation realism	6.76 (2.27)

Face and content validity were analysed descriptively, as these domains reflect subjective expert judgement rather than inferential hypotheses, consistent with prior simulator validation methodology.

### Content validity

Content validity was strongly endorsed, with 96%–97% of participants supporting the simulator's value for procedural training, pre‐training for novices, and integration into residency curricula (Table 5). Nearly all respondents agreed that simulator‐based training should be implemented before performing real procedures. In particular, 97.4% supported incorporating the simulator into structured residency training.

**Table 5:** Results of the questionnaire for experience with the simulator.

Domain/group	Novices	Intermediates	Experts	All groups
Potential to evolve	39/40 (97.4%)	20/20 (100%)	17/18 (94.4%)	76/78 (97.4%)
Useful procedural training	39/40 (97.4%)	19/20 (95%)	17/18 (94.4%)	75/78 (96.2%)
Pre‐training for all novices	39/40 (97.4%)	20/20 (100%)	17/18 (94.4%)	76/78 (97.4%)
Obligatory for novices before performing	35/40 (87.2%)	16/20 (80%)	14/18 (77.8%)	65/78 (83.3%)
Recommended to resident to improve skills	39/40 (97.4%)	19/20 (95%)	16/18 (88.9%)	74/78 (94.9%)
Integrated into curriculum	39/40 (97.4%)	20/20 (100%)	17/18 (94.4%)	76/78 (97.4%)

### Educational value and user‐friendliness

Educational value ratings were overwhelmingly positive: 97.4% agreed that the simulator was beneficial for skill training (Educational Value I), and 92.3% agreed it improved preparedness for real procedures (Educational Value II). In contrast, user‐friendliness received lower approval (35.9%), with common feedback citing stiffness of joint manipulation and the need for clearer guidance. Detailed frequencies are presented in Table 6.

**Table 6:** Results of the questionnaire for educational value and user‐friendliness, % (yes, no).

Domain/group	Intermediates	Experts	Intermediates + experts
Educational Value I	20/20 (100%)	18/19 (94.7%)	38/39 (97.4%)
Educational Value II	19/20 (95%)	17/19 (89.5%)	36/39 (92.3%)
User‐friendliness	8/20 (40%)	6/19 (31.2%)	14/39 (35.9%)

User‐friendliness concerns primarily reflected hardware‐related factors such as joint stiffness and resistance, rather than deficiencies in educational design or task structure.

## DISCUSSION

### Main findings

This study demonstrated strong construct, face and content validity for the tactile knee simulator. Experts completed tasks significantly faster than intermediates and novices on initial attempts (*p* < 0.001; *r* = 0.71–0.78), confirming clear performance separation between experience levels. All groups improved with repetition, with novices demonstrating the steepest early gains (292 s → 73 s), supporting rapid early‐phase learning.

Between‐group differences narrowed in later trials, although expert–intermediate differences remained significant at Trial 5 (*p* = 0.012; *r* = 0.47). However, interpretation of Trials 4 and 5 should be made cautiously, as only six novices completed Trial 5, and the apparent convergence likely reflects attrition of slower participants rather than true equivalence in skill levels. Performance curves plateaued after Trial 3 across groups, suggesting that the primary learning effect occurs within the first three repetitions rather than during later endurance‐based attempts.

Face and content validity were strongly endorsed (mean realism scores >7/10; 92%–97% curricular integration), while lower user‐friendliness ratings (35.9% agreement) highlight opportunities for ergonomic and instructional refinement. Persistent expert–intermediate separation in later trials may reflect both construct validity and ceiling effects inherent to a finite diagnostic navigation task. Specifically, once anatomical landmarks were consistently identified within minimal time thresholds, further discrimination between higher experience levels may have been constrained by task simplicity rather than true equivalence of arthroscopic competence.

### Comparison with existing simulators

These findings align with validation studies of existing arthroscopy simulators—including Simendo, ArthroS, and PASSPORT—which similarly demonstrated novice–expert performance separation [4, 10, 12, 13]. Systematic reviews and meta‐analyses likewise support simulation‐based training in improving surgical performance and supporting competency‐based orthopaedic curricula [7, 15]. High visual realism ratings in our study parallel those reported for shoulder and knee arthroscopy simulators [2, 14].

Distinct from most VR systems, the tactile knee incorporates physical haptics and dynamic joint manipulation, replicating triangulation and instrument resistance—capabilities often limited in VR platforms. Although cadaveric training remains the gold standard for anatomical realism, cost, ethical constraints and limited access restrict its scalability; physical simulators therefore offer a feasible, reusable alternative for early‐stage skill acquisition. Compared with cadaveric training, physical simulators offer advantages in cost, scalability, and repeated access, supporting their use in early‐stage training curricula. In addition, physical simulators avoid the logistical constraints, recurring costs and ethical considerations associated with cadaveric laboratories, allowing for repeated unsupervised practice within institutional skills centres.

### Educational implications

The simulator provided effective early‐phase arthroscopy training, enabling repeated practice of scope control and anatomical landmark recognition. The steep improvement trajectory among novices supports its value for onboarding and structured pre‐clinical skills development, while persistent expert–intermediate separation suggests potential use for competency benchmarking. User‐friendliness ratings were lower (35.9% agreement), indicating opportunities for ergonomic refinement and clearer instructional guidance. These usability considerations may reflect challenges related to joint manipulation mechanics and instrument handling.

### Limitations

This study has several limitations. First, its single‐centre design and convenience sampling may limit generalisability. Second, face and content validity relied on subjective questionnaire responses, introducing potential response bias. Third, the study did not include longitudinal follow‐up or comparison with operative performance, meaning transfer and predictive validity remain untested. Substantial attrition beyond Trial 3 also restricts the interpretation of late‐trial performance data, particularly among novices. Finally, the simulator lacks automated performance metrics and currently supports only diagnostic tasks.

Future research should evaluate whether simulator‐acquired skills translate into improved operative performance through blinded OSATS assessment in cadaveric or clinical environments. Expansion of modules to procedural content (e.g., meniscal debridement and ACL probing), integration of real‐time feedback, automated tracking metrics and multi‐institutional studies would enhance educational robustness and support curriculum‐wide adoption. It is noted that the evaluated task assesses navigational efficiency rather than comprehensive arthroscopic procedural competence.

Additionally, assessors were not blinded to participant experience level, introducing potential measurement bias. Accordingly, the findings should be interpreted as validating navigational efficiency and spatial orientation rather than comprehensive procedural or decision‐making.

## CONCLUSION

The tactile knee simulator demonstrated strong early‐phase construct, face and content validity for diagnostic knee arthroscopy training. It reliably discriminated between experience levels (*p* < 0.001) with large expert–novice effect sizes and showed substantial performance improvement across groups, with the steepest early gains observed in novices and the most consistent progression in intermediates. Realism and educational value were highly endorsed, although lower usability ratings highlight opportunities for ergonomic and instructional refinement. Future studies should evaluate transfer validity and long‐term operative performance outcomes to inform the simulator's potential integration within competency‐based training programs.

## AUTHOR CONTRIBUTIONS

Oliver Lim Tze Rong, Jeremy Lim Tze En and Fang Jingyan conceptualised and drafted the manuscript. Dileepa Banagala, Samundeeswari Saseendar, Salim AL Habsi, James Hui and Mok Ying Ren supervised the study design and execution. Dave Lee served as the principal investigator and corresponding author, overseeing all aspects of the research. All authors reviewed and approved the final manuscript.

## CONFLICT OF INTEREST STATEMENT

The authors declare no conflicts of interest.

## ETHICS STATEMENT

This study was approved by the National Healthcare Group Domain Specific Review Board (NHG DSRB), Singapore (approval number: 2024‐3242).

## Supporting information

Appendix SA.

Appendix SB.

## Data Availability

Data supporting the findings of this study are available from the corresponding author upon reasonable request.

## References

[1] Braman JP , Sweet RM , Hananel DM , Ludewig PM , Van Heest AE . Development and validation of a basic arthroscopy skills simulator. Arthroscopy. 2015;31(1):104–112. 10.1016/j.arthro.2014.07.012 25239171

[2] Cannon WD , Garrett Jr, WE , Hunter RE , Sweeney HJ , Eckhoff DG , Nicandri GT , et al. Improving residency training in arthroscopic knee surgery with use of a virtual‐reality simulator: a randomized blinded study. J Bone Joint Surg Am. 2014;96(21):1798–1806. 10.2106/JBJS.N.00058 25378507

[3] Cychosz C , Khazi ZM , Karam M , Duchman K , Willey M , Westermann R . Validation of a novel hip arthroscopy simulator: establishing construct validity. J Hip Preserv Surg. 2019;6(4):385–389. 10.1093/jhps/hnz059 PMC6990386 32015891

[4] Garfjeld Roberts P , Guyver P , Baldwin M , Akhtar K , Alvand A , Price AJ , et al. Validation of the updated ArthroS simulator. Knee Surg Sports Traumatol Arthrosc. 2017;25(2):616–625. 10.1007/s00167-016-4114-1 27085361

[5] Huri G , Gülşen MR , Karmiş EB , Karagüven D . Cadaver versus simulator‐based arthroscopic training in shoulder surgery. Turk J Med Sci. 2021;51(3):1179–1190. 10.3906/sag-2011-71 PMC8283431 33421972

[6] Monje Gil F , Hernandez Vila C , Moyano Cuevas JL , Lyra M , Pagador JB , Sanchez Margallo FM . Validation of a simulator for temporomandibular joint arthroscopy. Int J Oral Maxillofac Surg. 2016;45(7):836–841. 10.1016/j.ijom.2016.01.010 26850940

[7] Polce EM , Kunze KN , Williams BT , Krivicich LM , Maheshwer B , Beletsky A , et al. Efficacy and validity of orthopaedic simulators in surgical training: a systematic review and meta‐analysis of randomized controlled trials. J Am Acad Orthop Surg. 2020;28(24):1027–1040. 10.5435/JAAOS-D-19-00839 32398408

[8] Rahm S , Germann M , Hingsammer A , Wieser K , Gerber C . Validation of a virtual‐reality‐based simulator for shoulder arthroscopy. Knee Surg Sports Traumatol Arthrosc. 2016;24(5):1730–1737. 10.1007/s00167-016-4022-4 26860098

[9] Srivastava S , Youngblood PL , Rawn C , Hariri S , Heinrichs WL , Ladd AL . Initial evaluation of a shoulder arthroscopy simulator: establishing construct validity. J Shoulder Elbow Surg. 2004;13(2):196–205. 10.1016/j.jse.2003.12.009 14997099

[10] Stunt JJ , Kerkhoffs GMMJ , Horeman T , van Dijk CN , Tuijthof GJM . Validation of the PASSPORT V2 training environment for arthroscopic skills. Knee Surg Sports Traumatol Arthrosc. 2016;24(6):2038–2045. 10.1007/s00167-014-3213-0 25103120

[11] Tronchot A , Berthelemy J , Thomazeau H , Huaulmé A , Walbron P , Sirveaux F , et al. Performance discrimination using a virtual‐reality arthroscopy simulator. Orthop Traumatol Surg Res. 2021;107(8):103079. 10.1016/j.otsr.2021.103079 34597826

[12] Tuijthof GJM , Visser P , Sierevelt IN , van Dijk NC , Kerkhoffs GMMJ . Does perception of usefulness of arthroscopic simulators differ with levels of experience? Clin Orthop Relat Res. 2011;469(6):1701–1708. 10.1007/s11999-011-1797-y PMC3094627 21290203

[13] van der Heijden LLM , Reijman M , van der Steen MC , Janssen RPA , Tuijthof GJM . Validation of Simendo Knee Arthroscopy Virtual Reality Simulator. Arthroscopy: The Journal of Arthroscopic & Related Surgery. 2019;35(8):2385–2390. 10.1016/j.arthro.2019.01.031 31053458

[14] Waterman BR , Martin KD , Cameron KL , Owens BD , Belmont Jr, PJ . Simulation training improves surgical proficiency and safety during diagnostic shoulder arthroscopy performed by residents. Orthopedics. 2016;39(3):e479–e485. 10.3928/01477447-20160427-02 27135460

[15] Woodward CJ , Khan O , O'Neill N , Dasgupta P , Sinha J . Simulation‐based training in orthopaedic surgery: a systematic review. Curr Probl Surg. 2024;63:101676. 10.1016/j.cpsurg.2024.101676 39922638

