# Supplementary material for: Validation of a high‐fidelity simulator for diagnostic knee arthroscopy training
Source: J Exp Orthop. 2026 Mar 18;13(1):e70684. doi: 10.1002/jeo2.70684 (PMC13679890; doi:10.1002/jeo2.70684)
Supplement: Supplementary file 1 [file JEO2-13-e70684-s001.docx]

**Appendix A - Task Sequence for Simulator Trials**

| Position of Leg | Points to Probe |
| --- | --- |
| Knee in Flexion     | Insert Scope and begin identifying:     1. ACL (tibia footprint) 2. Medial femoral condyle 3. Medial tibial plateau 4. Lateral femoral condyle 5. Lateral tibial plateau |
| Extend knee and apply valgus force against side post     | 6. Medial Meniscus - Posterior Horn 7. Medial Meniscus - Mid Section |
| Apply varus force (Figure of 4) by placing in leg holder     | 8. Lateral Meniscus - Posterior Horn 9. Lateral Meniscus - Mid Section               Return to Flexion and Repeat    [Post Hoc END time for 9 Point validation] |
