# Supplementary material for: Validation of a high‐fidelity simulator for diagnostic knee arthroscopy training
Source: J Exp Orthop. 2026 Mar 18;13(1):e70684. doi: 10.1002/jeo2.70684 (PMC13679890; doi:10.1002/jeo2.70684)
Supplement: Supplementary file 2 [file JEO2-13-e70684-s002.docx]

**Appendix B – Questionnaire Items**

B1. Face validity items

| Face validity aspect | Question |
| --- | --- |
| Outer appearance | What is your opinion of the outer appearance of this simulator?  (Select a number from 1 to 10 - 1 being poor impression, 10 being good impression)  1 2 3 4 5 6 7 8 9 10    Is it clear in which joint you will be operating?  (Select a number from 1 to 10 - 1 being very unclear, 10 being very clear)  1 2 3 4 5 6 7 8 9 10    Is it clear which portals are being used?  (Select a number from 1 to 10 - 1 being very unclear, 10 being very clear)  1 2 3 4 5 6 7 8 9 10 |
| Intra-articular joint | How realistic is the intra-articular anatomy?  (Select a number from 1 to 10 - 1 being not realistic, 10 being very realistic)  1 2 3 4 5 6 7 8 9 10    How realistic is the texture of the structures?  (Select a number from 1 to 10 - 1 being not realistic, 10 being very realistic)  1 2 3 4 5 6 7 8 9 10    How realistic is the color of the structures?  (Select a number from 1 to 10 - 1 being not realistic, 10 being very realistic)  1 2 3 4 5 6 7 8 9 10    How realistic is the size of the structures?  (Select a number from 1 to 10 - 1 being not realistic, 10 being very realistic)  1 2 3 4 5 6 7 8 9 10    How realistic is the size of the intra-articular joint space?  (Select a number from 1 to 10 - 1 being not realistic, 10 being very realistic)  1 2 3 4 5 6 7 8 9 10    How realistic is the arthroscopic image?  (Select a number from 1 to 10 - 1 being not realistic, 10 being very realistic)  1 2 3 4 5 6 7 8 9 10    How realistic is the manipulation of the knee?  (Select a number from 1 to 10 - 1 being not realistic, 10 being very realistic)  1 2 3 4 5 6 7 8 9 10 |

B2. Educational value and user-friendliness

| Parameter | Question |
| --- | --- |
| Educational value I | The simulator allows training of joint inspection.  YES / NO |
| Educational value II | The simulator is a good way to prepare for a real-life arthroscopic operation.  YES / NO |
| User-friendliness | I felt the need to read a manual before operating the simulator.  YES / NO |

B3. Simulator perception questions

1. Do you think the Tactile simulators have potential to evolve as a training modality?

YES / NO

2. Do you think the Tactile simulators are useful for procedural training of diagnostic arthroscopy?

YES / NO

3. Do you think training on the Tactile simulators should be offered to all novices for pre-training before performing surgery on real patients?

YES / NO

4. Do you think the Tactile simulators training should be obligatory for pre-training novices before performing surgery on real patients?

YES / NO

5. Do you think the Tactile simulators training should be recommended for any orthopaedic resident to improve his/her skills individually?

YES / NO

6. Should the Tactile simulators training be integrated into the current curriculum of the specialization program of orthopedic surgeons?

YES / NO
